# Thyroid dysfunction and pregnancy outcomes

**Published:** 2015-07

**Authors:** Sima Nazarpour, Fahimeh Ramezani Tehrani, Masoumeh Simbar, Fereidoun Azizi

**Affiliations:** 1 *Department of Reproductive Health, Faculty of Nursing and Midwifery, Shahid Beheshti University of Medical Sciences, Tehran, Iran.*; 2 *Reproductive Endocrinology Research Center, Research Institute for Endocrine Sciences, Shahid Beheshti University of Medical Sciences, Tehran, Iran.*; 3 *Endocrine Research Center, Research Institute for Endocrine Sciences, Shahid Beheshti University of Medical Sciences, Tehran, Iran.*

**Keywords:** *Thyroid disease*, *Pregnancy outcome*, *Hypothyroidism*, *Hyperthyroidism*

## Abstract

**Background:**

Pregnancy has a huge impact on the thyroid function in both healthy women and those that have thyroid dysfunction. The prevalence of thyroid dysfunction in pregnant women is relatively high.

**Objective:**

The objective of this review was to increase awareness and to provide a review on adverse effect of thyroid dysfunction including hyperthyroidism, hypothyroidism and thyroid autoimmune positivity on pregnancy outcomes.

**Materials and Methods:**

In this review, Medline, Embase and the Cochrane Library were searched with appropriate keywords for relevant English manuscript. We used a variety of studies, including randomized clinical trials, cohort (prospective and retrospective), case-control and case reports. Those studies on thyroid disorders among non-pregnant women and articles without adequate quality were excluded.

**Results:**

Overt hyperthyroidism and hypothyroidism has several adverse effects on pregnancy outcomes. Overt hyperthyroidism was associated with miscarriage, stillbirth, preterm delivery, intrauterine growth retardation, low birth weight, preeclampsia and fetal thyroid dysfunction. Overt hypothyroidism was associated with abortion, anemia, pregnancy-induced hypertension, preeclampsia, placental abruption, postpartum hemorrhage, premature birth, low birth weight, intrauterine fetal death, increased neonatal respiratory distress and infant neuro developmental dysfunction. However the adverse effect of subclinical hypothyroidism, and thyroid antibody positivity on pregnancy outcomes was not clear. While some studies demonstrated higher chance of placental abruption, preterm birth, miscarriage, gestational hypertension, fetal distress, severe preeclampsia and neonatal distress and diabetes in pregnant women with subclinical hypothyroidism or thyroid autoimmunity; the other ones have not reported these adverse effects.

**Conclusion:**

While the impacts of overt thyroid dysfunction on feto-maternal morbidities have been clearly identified and its long term impact on childhood development is well known, data on the early and late complications of subclinical thyroid dysfunction during pregnancy or thyroid autoimmunity are controversial. Further studies on maternal and neonatal outcomes of subclinical thyroid dysfunction maternal are needed.

## Introduction

Thyroid hormones have profound variation during the life span and are associated with severe adverse health impacts (1, 2). Pregnancy, as an important reproductive event, has a profound but reversible effect on the thyroid gland and its functions. Pregnancy is actually a state of excessive thyroid stimulation leading to an increase in thyroid size by 10% in iodide sufficient areas and 20-40% in iodide deficient regions (3). Furthermore following the physiological and hormonal changes caused by pregnancy and human chorionic gonadotropin (HCG) the production of thyroxin (T4) and triiodothyronine (T3) increase up to 50% leading to 50% increase in a woman’s daily iodide need, while Thyroid-stimulating hormone (TSH) levels are decreased, especially in first trimester (4). In an iodide sufficient area, these thyroid adaptations during pregnancy are well tolerated, as stored inner thyroid iodide is enough; however in iodide deficient areas, these physiological adaptations lead to significant changes during pregnancy (5). 

Furthermore in women who suffered from thyroid dysfunction prior to pregnancy, the hormonal changes mentioned are magnified, leading to possibly adverse pregnancy outcomes if not been treated appropriately. Furthermore the mode of delivery may additionally have adverse impact on fetal- pituitary- thyroid axis (6). The prevalence of thyroid dysfunction in pregnant women is relatively high so that overt thyroid dysfunction occurs in 2-3% of pregnancies, and subclinical dysfunction in 10% of pregnancies (7) and thyroid autoimmunity is even more prevalent (8).

Given the high prevalence of thyroid disturbances in pregnancy and lack of adequate review article summarizing the effect of thyroid dysfunction on pregnancy and neonatal outcomes, we aimed to summarize the adverse effects of thyroid dysfunction including hyperthyroidism, hypothyroidism and thyroid autoimmune positivity on pregnancy outcomes. Research question was: "are thyroid disorders in pregnant women associated with adverse effects on pregnancy outcomes"?

**Evidence acquisition**

This review study was conducted with a prospective protocol. We searched Medline (1985-2013), Embase (1985-2013) and the Cochrane Library (2012) for relevant English manuscripts. Using keywords including “thyroid”, “thyroid dysfunction”, “thyroid disorder”, “hyperthyroidism”, “hypothyroidism”, “euthyroidism”, “subclinical hypothyroidism”, “subclinical hyperthyroidism”, “thyroid autoantibodies”, “pregnancy outcome”, “miscarriage”, “abortion”, “pregnancy loss”, “preterm”, “premature”, “early labor”, “Thyroid peroxidase” and “cognitive” to generate a subset of citations relevant to our research question. Subclinical hypothyroidism (SCH) is defined as a serum TSH level above the upper limit of normal despite normal levels of serum free thyroxine and subclinical hyperthyroidism is defined as serum thyroid hormone levels within their respective reference ranges in the presence of low-undetectable serum TSH levels (9). Overt hypothyroidism is defined as a serum free T4 level lower than the upper limit of normal and overt hyperthyroidism is defined as a serum free T4 level above the upper limit of normal. Thyroid autoimmunity is defined as increase in thyroid auto antibodies above the upper limit of normal with or without thyroid disturbances. 

The full manuscripts of all citations that met our study objective were selected and obtained. In cases of duplicate publications, we selected the most recent and complete versions. From the 4480 citations identified from electronic searches, at the beginning, we found 512 related articles; 130 studies on overt hypothyroidism, 203 on subclinical hypothyroidism, 69 on overt hyperthyroidism, 43 on subclinical hyperthyroidism, and 67 on thyroid immunity. Of these articles, 58 met our study objectives, including 11 on hyperthyroidism, 22 on hypothyroidism and 26 on thyroid immunity.

We included all qualified original articles on our study subject; including randomized clinical trials, cohort (prospective and retrospective), case-control and case reports. We excluded non-English manuscript, those conducted on non-pregnant women and those with poor quality methodology. The titles and abstracts of all of the studies were evaluated by two non-dependent persons and those met inclusion criteria were appraised.

**Figure 1:**
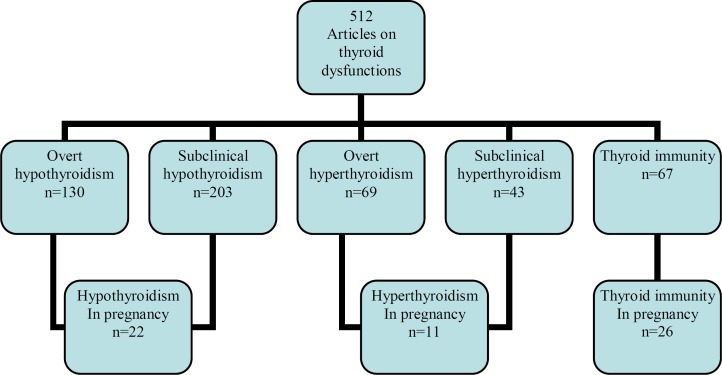
The number of articles that were reviewed in the study

## Results

**Hyperthyroidism and its adverse pregnancy and neonatal outcomes**

The natural physiological changes during pregnancy can mimic some of the signs observed in hyperthyroidism, including increased in basal metabolism, heart rate, fatigue, anxiety, palpitations, heat intolerance, warm and wet skin, hand tremors and systolic murmur; as a result the diagnosis of hyperthyroidism during pregnancy could cause clinical difficulties (9-11). Pregnant women, who suffer from hyperthyroidism, have more severe tachycardia and thyromegaly, along with exophthalmia, and lack of weight gain despite receiving adequate food (10).

Overt hyperthyroidism during pregnancy was not prevalent and was reported in 2 out of 1000 pregnancies (0.2%), while subclinical hyperthyroidism was occurred in 1.7% of pregnancies (11, 12). The most prevalent reason for hyperthyroidism during pregnancy was the transient hyperthyroidism resulting from hyperemesis gravidarum (THHG) due to the thyroid stimulation of beta-HCG (13); it was more prevalent in Asian populations compared to Europeans (14).

Except for THHG, the etiologies of thyrotoxicosis during pregnancy are the same as for non-pregnant women; it is most prevalent in Grave’s Disease caused by thyrotropin receptor antibodies stimulating the thyroid (TRabs) (11, 15). It is well documented that overt hyperthyroidism has several adverse effects on pregnancy outcomes, e.g. miscarriage, stillbirth, preterm delivery, intrauterine growth retardation, preeclampsia (11, 16). Furthermore women with Graves' disease have antibodies that can stop or stimulate the fetal anti-TSH receptor of thyroid gland (15, 17, 18).

There is no consensus regarding the adverse effect of subclinical hypothyroidism on pregnancy or neonatal outcomes. Casey et al*. *reported no significant increase of placenta abruption, preterm labor and low birth weight in pregnancy complicated by subclinical hyperthyroidism in comparison with euthyroid ones (19). Table I summarizes the results of the most relevant studies on the impact of overt and subclinical hyperthyroidism on pregnancy and neonatal outcomes.

**Hypothyroidism and its adverse pregnancy and fetal outcomes**

Pregnancy can imitate some of the signs that are observed in hypothyroidism, including fatigue, anxiety, constipation, muscle cramps, and weight gain; as a result, the clinical diagnosis of hypothyroidism during pregnancy may be difficult (10, 27). Moreover, most signs of hypothyroidism can be hidden by a woman’s status following the increase in metabolism in pregnancy. Furthermore the thyroid hormonal profile in normal pregnancy can be mis-interoperated as hypothyroidism and as a result the interpretation of thyroid function tests needs trimester-specific reference intervals for a specific population (14, 15). Applying trimester-specific reference ranges of thyroid hormones prevents misclassification of thyroid dysfunction during pregnancy. Compared to hyperthyroidism, hypothyroidism is very common during pregnancy; 2-3% of pregnant women suffer from hypothyroidism (0.3-0.5% overt hypothyroidism and 2-2.5% subclinical hypothyroidism) (11, 28).

While the main etiology for hypothyroidism during pregnancy worldwide is iodide insufficiency, however in iodide sufficient areas its main cause is autoimmune thyroiditis (8). SCH is the most common thyroid dysfunction during pregnancy (11, 29). Its prevalence varies between 1.5-5% based on various definitions, different ethnicity, iodine consumption and nutrition life style as well as study designs (30). While the adverse effects of SCH accompanied with positive TPO antibodies or overt hypothyroidism on pregnancy outcome are well known, however there is controversy on negative impact of SCH without autoimmunity on pregnancy outcomes (27, 31-33). Pregnant women that possess the TPO antibodies during the initiation of their pregnancy are subjected to subclinical hypothyroidism during their pregnancy or thyroid dysfunction after childbirth (12).

Table II and III summarize the studies on adverse outcomes of overt and subclinical hypothyroidism, respectively. As it has been shown the overt hypothyroidism is associated with increase in prevalence of abortion, anemia, pregnancy-induced hypertension, preeclampsia, placental abruption, postpartum hemorrhage, premature birth, low birth weight, intrauterine fetal death and neonatal respiratory distress (15, 27, 29, 32, 34-41).

There is no consensus on adverse impacts of subclinical hypothyroidism on pregnancy outcomes; while some studies demonstrated higher chance of placental abruption, preterm birth, miscarriage, gestational hypertension, fetal distress, severe preeclampsia and neonatal distress and diabetes, the other study have not reported and adverse effect (31, 33, 42, 43, 45, 46). The long term effect of overt hypothyroidism on cognitive function has been well documented; these children have lower IQ and more developmental dysfunction (8, 12, 15, 38-41, 46-48), however there is no consensus on the long term cognitive effects of subclinical hypothyroidism; while some reported loss of motor function and intelligence in infants and children the other reported a normal motor and cognitive function (15, 45, 48, 50, 51). Table IV summarizes the cognitive function of infants and children been affected by overt or subclinical hypothyroidism during pregnancy.

**Autoimmune thyroid disorders**

Anti-thyroid antibodies are relatively common among women during their reproductive ages, 6-20% of all euthyroid women are positive for anti-thyroid antibodies (8). The presence of anti-thyroid antibodies during a woman’s reproductive age is not necessarily followed by a thyroid dysfunction and 10-20% of all pregnant women who are TPO antibody positive remain euthyroid in first trimester (3, 56). Despite the high prevalence of TPO antibody positive among reproductive age women, there is no consensus on the feto-maternal complications of euthyroid pregnant women who are TPO antibody positive. As a result, routine screening of pregnant women for thyroid antibodies is controversial (57, 58).

Table 7 summarizes the results of the most relevant studies regarding the feto-maternal outcomes of thyroid autoimmune positivity in euthyroid women. While adverse outcomes such as abortion, preterm delivery, recurrent miscarriage, hypertension, fetal dyspenea and diabetes are reported in some of studies, other studies report compatible pregnancy outcomes (27, 40, 44, 45, 46, 59-66). Furthermore Ghassabian and Tiemeier showed that the high titration of anti-thyroid peroxidase antibodies (TPO-Ab) during pregnancy associated with an increased risk of cognitive and behavioral problems in preschool children (67).

**Table I:** The adverse effects of hyperthyroidism (overt/subclinical) on pregnancy and neonatal outcomes

**Authors**	**Year of publication**	**Location**	**Type of study**	**Participants**	**Outcome**
**Overt hyperthyroidism**					
Davis et al. (20)	1989	USA	Prospective	60 Pregnant women with overt thyrotoxicosis	small for gestational age births, stillbirths, and possibly congenital malformations
Kriplani et al. (21)	1994	India	Prospective	32 pregnancies complicated by hyperthyroidism	Preterm labor, pregnancy induced hypertension thyroid crisis, intrauterine growth retardation. Abnormal Thyroid status of neonates.
Millar et al. (22)	1994	USA	Retrospective	181 hyperthyroid pregnant women	Low birth weight infants and severe preeclampsia.
Phoojaroenchanachai et al. (23)	2001	Thailand	Retrospective	293 pregnant women with present and past history of hyperthyroidism	Low birth weight
Peleg et al. (18)	2002	USA	Retrospective	Twenty-nine women with a history of Graves disease and positive thyroid-stimulating immunoglobulin	Neonatal thyrotoxicosis
Polak et al. (17)	2004	France	Prospective	72 pregnant women with a history of Graves' disease.	Fetal goiter
Luton et al. (24)	2005	France	prospective	72 pregnant women (72 fetuses)	One fetus had moderate hypothyroidism (1 fetus), goiter (11 fetuses at 32 weeks), and fetal thyroid dysfunction
Luewan et al. (25)	2011	Thailand	Prospective (cohort)	540 pregnant women (180 with hyperthyroidism and 360 controls)	Fetal growth restriction, preterm birth and low birth weight, tendency to have a higher rate of pregnancy-induced hypertension.
Männistö et al. (26)	2013	USA	Retrospective (cohort)	223512 singleton pregnancies	Preeclampsia, superimposed preeclampsia, preterm birth, induction, neonatal intensive-care unit admission
**Subclinical hyperthyroidism**					
Casey et al. (19)	2006	USA	Prospective (cohort)	A total of 25,765 women underwent thyroid screening and 433 women were considered to have subclinical hyperthyroidism	Subclinical hyperthyroidism is not associated with adverse pregnancy outcomes

**Table II:** The adverse effects of overt hypothyroidism on pregnancy outcomes

**Authors**	**Year of publication**	**Location**	**Type of study**	**Participants**	**Outcomes**
Abalovich et al (35)	2002	Argentina	Randomized Clinical Trial	114 women with primary hypothyroidism (16 overt hypothyroidism)	Abortion, premature delivery
Wolfberg et al (37)	2005	USA	Retrospective	19,969 women (482 with treated hypothyroid disease and 19,487 without thyroid disease)	Pre-eclampsia
Idris et al (36)	2005	England	Retrospective	167 pregnant women	Low birth weight caesarean section
Cleary Goldman et al (33)	2008	USA	Prospective	10,990 pregnant women	Preterm labor , macrosomia, gestational diabetes
Sahu et al (32)	2010	India	Prospective	633 pregnant women	Pregnancy-induced hypertension, intrauterine growth restriction, intrauterine demise, Neonatal complications, gestational diabetes
Hirsch et al (38)	2013	Israel	Retrospective case series	306 pregnant women (101 with hyperthyroidism and 205 euthyroid)	Abortions and premature delivery
Männistö et al (26)	2013	USA	Retrospective	223512 singleton pregnancies	**Primary hypothyroidism**: Preeclampsia, superimposed preeclampsia, gestational diabetes, preterm birth, induction , cesarean section, intensive-care unit admission **Iatrogenic hypothyroidism**: placental abruption , breech presentation, cesarean section after spontaneous labor

**Table III:** The adverse effects of subclinical hypothyroidism (with/without thyroid autoimmunity) on pregnancy outcomes

**Authors**	**Year of publication**	**Location**	**Type of the study**	**Participants**	**Outcomes**
**Subclinical hypothyroidism without control of TPOAb**					
Abalovich et al (35)	2002	Argentina	Prospective	114 women with primary hypothyroidism (35 subclinical hypothyroidism	Abortion, premature delivery
Stagnaro-Green et al (42)	2005	USA	Prospective (nested-case control)	953 women	Very preterm delivery
Casey et al (29)	2005	USA	Prospective	25,756 women	Placental abruption Preterm birth
Cleary-Goldman et al (33)	2008	USA	Prospective	10,990 patients	Subclinical hypothyroidism was not associated with adverse outcomes.
Sahu et al (32)	2010	India	Prospective	633 women	Cesarean section rate for fetal distress
Wilson et al (31)	2012	USA	Prospective	24,883 women	Severe preeclampsia
**Subclinical hypothyroidism including negative and positive TPO Ab**					
Negro et al (27)	2006	Italy	Randomized Clinical Trial	984 pregnant women	Pregnant women who are positive for TPOAb develop impaired thyroid function, increased risk of miscarriage and premature deliveries
Benhadi et al (43)	2009	Netherlands	prospective (cohort)	2497 women	Pregnant women without overt thyroid dysfunction, the risk of child loss increased with higher levels of maternal TSH
Karakosta et al (44)	2012	Greece	prospective	1170 pregnant women	Increased gestational diabetes and low birth weight neonates among those with of high TSH and spontaneous preterm among those without elevated TSH levels

**Table IV:** The cognitive function of infants and children been affected by overt or subclinical hypothyroidism during pregnancy

**Authors**	**Year of publication**	**Location**	**Type of the study**	**Participants**	**Result**
**Overt hypothyroidism**					
Liu et al (52)	1994	China			All children showed normal IQs
Haddow et al (39)	1999	England	Prospective (cohort)	25216 women	Adversely affect their children's subsequent performance on neuropsychological tests.
Pop et al (47)	2003	Netherlands	Prospective	125 children of women with hypothyroxinaemia (63 cases and 62 controls)	Delay in infant neurodevelopment.
Kooistra et al (48)	2006	Netherlands	Retrospective (case control)	204 (108 neonates who were born to mothers with hypothyroidism and 96 control)	Lower scores on the Neonatal Behavioral Assessment Scale and orientation index
Li et al (50)	2010	China	Prospective (cohort)	213 (18 isolated subclinical hypothyroidism, 19 hypothyroxinaemia, 34 euthyroid TPOAb positive and 142 controls)	Lower motor and intellectual development at 25-30 months.
Henrichs et al (49)	2010	Netherlands	Prospective (Population-based cohort)	3659 children and their mothers	Higher risk of expressive language and nonverbal cognitive delay
Chevrier et al (53)	2011	USA	Prospective (cohort)	287 pregnant women and their children	No adverse effect on child neurodevelopment.
Downing et al (54)	2012	USA	Case report	Three women with hypothyroidism	Children had average or above average results on all parameters. Comparative scores of the neuropsychological tests in sibling pairs for full-scale IQ and performance IQ were variable; some scores were higher and some lower in children with congenital hypothyroidism.
Momotani et al (55)	2012	Japan	Case report	Five women with overt hypothyroidism	The development scores (the Tsumori-Inage Infant's Developmental Test or the Wechsler Intelligence Scale) of all the children turned out to be either normal or advanced.
**Subclinical hypothyroidism**					
Li et al (50)	2010	China	Prospective (cohort)	213 (18 isolated subclinical hypothyroidism, 19 hypothyroxinaemia , 34 euthyroid TPOAb positive and 142 controls)	Lower motor and intellectual development at 25-30 months.
Ghorbani Behrooz et al (51)	2012	Iran	Prospective (Historical cohort)	62 children of mothers who had subclinical hypothyroidism	No adverse effect on IQ level and cognitive performance of children

**Table V:** The feto-maternal outcomes of thyroid autoimmune positivity in euthyroid pregnant women

**Authors**	**Year of publication**	**Location**	**Type of Study**	**Participants**	**Outcome**
Stagnaro-Green et al (68)	1990	USA	Prospective	552 pregnant women	Increased miscarriage
Glinoer et al (40)	1991	Belgium	Prospective	120 euthyroid pregnant women	Increased spontaneous abortion
Pratt et al (64)	1993	USA	Retrospective (case control)	45 women and 100 apparently health blood donors served as controls.	Increased recurrent spontaneous abortions
Glinoer et al (41)	1994	Belgium	Prospective	87 healthy pregnant women with thyroid antibodies and normal thyroid function	Increased spontaneous miscarriage and premature deliveries
Bussen et al (63)	1995	Germany	Retrospective	66 women (22 euthyroid non-pregnant habitual aborters; 22 nulligravidae and 22 multigravidae without endocrine dysfunction as controls).	Increased habitual abortions
Singh et al (69)	1995	USA	Retrospective	487 subfertile women who had undergone Assisted reproductive technology	Increased miscarriage
Iijima et al (70)	1997	Japan	Prospective	1, 179 healthy pregnant women including 228 cases of positive thyroid autoantibody	Increased spontaneous abortion
Kutteh et al (62)	1999	USA	Retrospective.	1588 women (700 women with a history of pregnancy losses, 688 women with a history of infertility who were undergoing Assisted reproductive technology, and 200 healthy, reproductive-aged female controls)	Increased recurrent pregnancy loss
Muller et al (66)	1999	Netherlands	Prospective (nested-case control)	173 subfertile women undergoing Invitro fertilization	No increase of miscarriage in women without a history of habitual abortion
Dendrinos et al (60)	2000	Greece	Retrospective (case control)	45 women (30 euthyroid with Recurrent spontaneous miscarriage and 15 matched controls)	Increased recurrent spontaneous miscarriage
Bagis et al (59)	2001	Turkey	Retrospective	876 women	Increased abortion
Poppe et al (71)	2003	Belgium	Prospective,	234 subfertile women undergoing Assisted reproductive technology	Increased miscarriage
Marai et al (72)	2004	Israel	Retrospective (case control)	66 women (58 with impaired fertility and 28 control parous women)	Increased recurrent miscarriages
Stagnaro-Green et al (42)	2005	USA	Prospective (nested-case control)	124 Cases and 124 Controls were randomly selected from among the 953 women who delivered at term	Increased very preterm delivery
Negro et al (27)	2006	Italy	Randomized Clinical Trial	984 pregnant women (TPO Ab + & -, euthyroid & subclinical)	Increased miscarriage and premature deliveries
Ghafoor et al (73)	2006	Pakistan	Prospective	1, 500 pregnant women	Increased low-birth-weight of neonates and high abortion rate
Negro et al (46)	2007	Italy	Retrospective	416 euthyroid women (42 were positive TPOAb) undergoing Assisted reproductive technology	Increased unsuccessful pregnancy or subsequent miscarriage
Iravani et al (74)	2008	Iran	Retrospective (case-control)	641 women with a history of 3 or more consecutive pregnancy losses and 269 controls	Increased recurrent abortion
Cleary-Goldman et al (33)	2008	USA	Prospective	10,990 pregnant women	Increased preterm premature rupture of membranes
Männistö et al (75)	2009	Finland	Prospective	9, 247 singleton pregnancies	Increased perinatal death
Soltanghoraee et al (76)	2010	Iran	Retrospective (case control)	95 cases as fertile controls and 70, 78 and 137 cases with infertility and recurrent abortion respectively.	Increased recurrent abortion
Haddow et al (77)	2010	USA	Prospective	10, 062 singleton pregnancies	Increased preterm delivery, premature rupture of membranes
Negro et al (78)	2011	Italy	Prospective	3593 pregnant women	Increased very preterm delivery and respiratory distress
Nambiar et al (61)	2011	India	Prospective	483 pregnant women	Increased miscarriage
Ashoor et al (65)	2011	Europe	Prospective	4, 420 singleton pregnancies	No increase spontaneous early preterm delivery
Karakosta et al (44)	2012	Greece	Prospective	1170 pregnant women (TPO Ab + & - , euthyroid & subclinical)	Increased gestational diabetes and low birth weight neonates among those with of high TSH and spontaneous preterm among those without elevated TSH levels

## Conclusion

Although it is well documented that overt hypothyroidism and overt hyperthyroidism have deleterious impacts on pregnancy and childhood outcomes, there is however no consensus on the potential impact of subclinical hypothyroidism and subclinical hyperthyroidism on maternal and fetal health. Furthermore there is debate on the association between miscarriage and preterm delivery in euthyroid women positive for TPO antibodies. As a result the universal screening of pregnant women has not been recommended yet, as the benefits of identification of those subclinical for of thyroid disturbances has not been proved. There is not adequate data on cost-benefit of treatment of pregnant women suffer from subclinical thyroid disorders. Studies are now focusing on these controversial issues to produce critically needed data on the impact of treating these subclinical forms of thyroid disease on the mother, fetus, and the future intellect of the unborn child. The present review article is limited by not including the non-English articles. Summarizing the studies which have been published, the following can be concluded: 1) Overt hyperthyroidism and hypothyroidism have several adverse effects on pregnancy outcomes, 2) The long term effect of overt hypothyroidism on cognitive function has been well documented, 3) There is debate on short and long term effect of subclinical hypothyroidism, 4) Thyroid antibody positivity is associated with adverse pregnancy outcomes, but there is no consensus on feto-maternal complication of pregnant women with TPO antibody positive and euthyroid status.

Future studies should include the following: 1) Studies of possible benefits of levo-T4 (L-T4) in euthyroid and subclinical hypothyroidism women with positive TPO antibody; 2) Larger randomized control trials of patients with maternal hypothyroidism are necessary to impact on neurocognitive function; 3) More comprehensive studies with controlled iodine intake checks (urinary tests, for example) are suggested.

## Note

***This article extracted from Ph.D. thesis. (Sima Nazarpour)***

## References

[1] Ramezani Tehrani F, Aghaee M, Asefzadeh S (2003). The comparison of thyroid function tests in cord blood following cesarean section or vaginal delivery. Int J Endocrinol Metab.

[2] Zadeh-Vakili A, Ramezani Tehrani F, Hashemi S, Amouzegar A, Azizi F (2012). Relationship between Sex Hormone Binding Globulin, Thyroid Stimulating Hormone, Prolactin and Serum Androgens with Metabolic Syndrome Parameters in Iranian Women of Reproductive Age. Diabetes Metabolism.

[3] The American Thyroid Association Taskforce on Thyroid Disease During Pregnancy and Postpartum (2011). Guidelines of the American Thyroid Association for the Diagnosis and Management of Thyroid Disease during Pregnancy and Postpartum. Thyroid.

[4] Yamamoto T, Amino N, Tanizawa O, Doi K, Ichihara K, Azukizawa M (1979). Longitudinal study of serum thyroid hormones, chorionic gonadotrophin and thyrotrophin during and after normal pregnancy. Clin Endocrinol (Oxf).

[5] Glinoer D, de Nayer P, Bourdoux P, Lemone M, Robyn C, van Steirteghem A (1990). Regulation of maternal thyroid function during pregnancy. J Clin Endocrinol Metab.

[6] Ramezani Tehrani F, Tohidi M, Rostami Dovom M, Azizi F (2011). A Population Based Study on the Association of Thyroid Status with Components of the Metabolic Syndrome. Diabete Metab.

[7] Azizi F, Delshad H (2014). Thyroid Derangements in Pregnancy. IJEM.

[8] Cignini p, Cafà EV, Giorlandino C, Capriglione S, Spata A, Dugo N (2012). Thyroid physiology and common diseases in pregnancy: review of literature. J Prenat Med.

[9] Casey B, Leveno K (2006). Thyroid disease in pregnancy. Obstet Gynecol.

[10] Cunningham F, Leveno KJ, Bloom SL, Hauth JC, Rouse Dj, Spong CY (2010). Williams Obstetrics.

[11] Delshad H, Azizi F (2008). Thyroid and pregnancy. J Med Council Iran.

[12] Banerjee S (2011). Thyroid Disorders in Pregnancy. JAPI.

[13] Glinoer D, Spencer CA (2010). Serum TSH determinations in pregnancy: how, when and why?. Nat Rev Endocrinol.

[14] Lazarus JH (2011). Thyroid function in pregnancy. Br Med Bul.

[15] El Baba KA, Azar ST (2012). Thyroid dysfunction in pregnancy. Int J Gen Med.

[16] Gaberšček S, Zaletel K (2011). Thyroid physiology and autoimmunity in pregnancy and after delivery. Expert Rev Clin Immunol.

[17] Polak M, Le Gac I, Vuillard E, Guibourdenche J, Leger J, Toubert ME (2004). Fetal and neonatal thyroid function in relation to maternal Gravesʼ disease. Best Pract Res Clin Endocrinol Metab.

[18] Peleg D, Cada S, Peleg A, Ben-Ami M (2002). The relationship between maternal serum Thyroid-stimulating immunoglobulin and fetal and neonatal thyrotoxicosis. Obstet Gynecol.

[19] Casey BM, Dashe JS, Wells CE, McIntire DD, Leveno KJ, Cunningham FG (2006). Subclinical hyperthyroidism and pregnancy outcomes. Obstet Gynecol.

[20] Davis LE, Lucas MJ, Hankins GD, Roark ML, Cunningham FG (1989). Thyrotoxicosis complicating pregnancy. Am J Obstet Gynecol.

[21] Kriplani A, Buckshee K, Bhargava VL, Takkar D, Ammini AC (1994). Maternal and perinatal outcome in thyrotoxicosis complicating pregnancy. Eur J Obstet Gynecol Reprod Biol.

[22] Millar LK, Wing DA, Leung AS, Koonings PP, Montoro MN, Mestman JH (1994). Low birth weight and preeclampsia in pregnancies complicated by hyperthyroidism. Obstet Gynecol.

[23] Phoojaroenchanachai M, Sriussadaporn S, Peerapatdit T, Vannasaeng S, Nitiyanant W, Boonnamsiri V (2001). Effect of maternal hyperthyroidism during late pregnancy on the risk of neonatal low birth weight. Clin Endocrinol (Oxf).

[24] Luton D, Le Gac I, Vuillard E, Castanet M, Guibourdenche J, Noel M (2005). Management of Graves' disease during pregnancy: the key role of fetal thyroid gland monitoring. J Clin Endocrinol Metab.

[25] Luewan S, Chakkabut P, Tongsong T (2011). Outcomes of pregnancy complicated with hyperthyroidism: a cohort study. Arch Gynecol Obstet.

[26] Männistö T, Mendola P, Grewal J, Xie Y, Chen Z, Laughon SK (2013). Thyroid diseases and adverse pregnancy outcomes in a contemporary US cohort. J Clin Endocrinol Metab.

[27] Negro R, Formoso G, Mangieri T, Pezzarossa A, Dazzi D, Hassan H (2006). Levothyroxine treatment in euthyroid pregnant women with autoimmune thyroid disease: effects on obstetrical complications. J Clin Endocrinol Metab.

[28] Negro R, Mestman JH (2011). Thyroid disease in pregnancy. Best Pract Res Clin Endocrinol Metab.

[29] Casey BM, Dashe JS, Wells CE, McIntire DD, Byrd W, Leveno KJ (2005). Subclinical hypothyroidism and pregnancy outcomes. Obstet Gynecol.

[30] Vanderpump MPJ (2011). The epidemiology of thyroid disease. Br Med Bulletin.

[31] Wilson KL, Casey BM, McIntire DD, Halvorson LM, Cunningham FG (2012). Subclinical thyroid disease and the incidence of hypertension in pregnancy. Obstet Gynecol.

[32] Sahu MT, Das V, Mittal S, Agarwal A, Sahu M (2010). Overt and subclinical thyroid dysfunction among Indian pregnant women and its effect on maternal and fetal outcome. Arch Gynecol Obstet.

[33] Cleary-Goldman J, Malone FD, Lambert-Messerlian G, Sullivan L, Canick J, Porter TF (2008). Maternal thyroid hypofunction and pregnancy outcome. Obstet Gynecol.

[34] Vissenberg R, Goddijn M, Mol BW, van der Post JA, Fliers E, Bisschop PH (2012). Thyroid dysfunction in pregnant women: clinical dilemmas. Ned Tijdschr Geneeskd.

[35] Abalovich M, Gutierrez S, Alcaraz G, Maccallini G, Garcia A, Levalle O (2002). Overt and Subclinical Hypothyroidism Complicating Pregnancy. Thyroid.

[36] Idris I, Srinivasan R, Simm A, Page RC (2005). Maternal hypothyroidism in early and late gestation: effects on neonatal and obstetric outcome. Clin Endocrinol.

[37] Wolfberg AJ, Lee-Parritz A, Peller AJ, Lieberman ES (2005). Obstetric and neonatal outcomes associated with maternal hypothyroid disease. J Matern Fetal Neonatal Med.

[38] Hirsch D, Levy S, Nadler V, Kopel V, Shainberg B, Toledano Y (2013). Pregnancy outcomes in women with severe hypothyroidism. Eur J Endocrinol.

[39] Haddow JE, Palomaki GE, Allan WC, Williams JR, Knight GJ, Gagnon J (1999). Maternal thyroid deficiency during pregnancy and subsequent neuropsychological development of the child. N Engl J Med.

[40] Glinoer D, Soto MF, Bourdoux P, Lejeune B, Delange F, Lemone M (1991). Pregnancy in patients with mild thyroid abnormalities: maternal and neonatal repercussions. J Clin Endocrinol Metab.

[41] Glinoer D, Riahi M, Grün JP, Kinthaert J (1994). Risk of subclinical hypothyroidism in pregnant women with asymptomatic autoimmune thyroid disorders. J Clin Endocrinol Metab.

[42] Stagnaro-Green A, Chen X, Bogden JD, Davies TF, Scholl TO (2005). The thyroid and pregnancy: a novel risk factor for very preterm delivery. Thyroid.

[43] Benhadi N, Wiersinga WM, Reitsma JB, Vrijkotte TG, Bonsel GJ (2009). Higher maternal TSH levels in pregnancy are associated with increased risk for miscarriage, fetal or neonatal death. Eur J Endocrinol.

[44] Karakosta P, Alegakis D, Georgiou V, Roumeliotaki T, Fthenou E, Vassilaki M (2012). Thyroid dysfunction and autoantibodies in early pregnancy are associated with increased risk of gestational diabetes and adverse birth outcomes. J Clin Endocrinol Metab.

[45] Stagnaro-Green A (2011). Thyroid antibodies and miscarriage: where are we at a generation later?. J Thyroid Res.

[46] Negro R, Formoso G, Coppola L, Presicce G, Mangieri T, Pezzarossa A (2007). Euthyroid women with autoimmune disease undergoing assisted reproduction technologies: the role of autoimmunity and thyroid function. J Endocrinol Invest.

[47] Pop VJ, Brouwers EP, Vader HL, Vulsma T, van Baar AL, Vijlder JJ (2003). Maternal hypothyroxinaemia during early pregnancy and subsequent child development: a 3-year follow-up study. Clin Endocrinol.

[48] Kooistra L, Crawford S, van Baar AL, Brouwers EP, Pop VJ (2006). Neonatal effects of maternal hypothyroxinemia during early pregnancy. Pediatrics.

[49] Henrichs J, Bongers-Schokking JJ, Schenk JJ, Ghassabian A, Schmidt HG, Visser TJ (2010). Maternal Thyroid Function during Early Pregnancy and Cognitive Functioning in Early Childhood: The Generation R Study. J Clin Endocrinol Metab.

[50] Li Y, Shan Z, Teng W, Yu X, Li Y, Fan Ch (2010). Abnormalities of maternal thyroid function during pregnancy affect neuropsychological development of their children at 25-30 months. Clin Endocrinol.

[51] Ghorbani Behrooz H, Tohidi M, Mehrabi Y, Ghorbani Behrooz E, Tehranidoost Mehdi, Azizi F (2012). Subclinical Hypothyroidism in Pregnancy: Intellectual Development of Offspring. Thyroid.

[52] Liu H, Momotani N, Noh JY, Ishikawa N, Takebe K, Ito K (1994). Maternal hypothyroidism during early pregnancy and intellectual development of the progeny. Arch Intern Med.

[53] Chevrier J, Harley KG, Kogut K, Holland N, Johnson C, Eskenazi B (2011). Maternal Thyroid Function during the Second Half of Pregnancy and Child Neurodevelopment at 6, 12, 24, and 60Months of Age. J Thyroid Res.

[54] Downing S, Halpern L, Carswell J, Brown RS (2012). Neuropsychological Development in Children of Mothers with Hypothyroidism Is Normal When Euthyroidism Is Achieved after Conception. Clin Thyroid.

[55] Momotani N, Iwama S, Momotani K (2012). Neurodevelopment in children born to hypothyroid mothers restored to normal thyroxine (T4) concentration by late pregnancy in Japan: no apparent influence of maternal T4 deficiency. J Clin Endocrinol Metab.

[56] van den Boogaard E, Vissenberg R, Land JA, van Wely M, van der Post JA, Goddijn M (2011). Significance of (sub)clinical thyroid dysfunction and thyroid autoimmunity before conception and in early pregnancy: a systematic review. Hum Reprod Update.

[57] Stagnaro-Green A, Abalovich M, Alexander E, Azizi F, Mestman J, Negro R (2011). Guidelines of the American Thyroid Association for the diagnosis and management of thyroid disease during pregnancy and postpartum. Thyroid.

[58] Kahric-Janicic N, Soldin SJ, Soldin OP, West T, Gu J, Jonklaas J (2007). Tandem mass spectrometry improves the accuracy of free thyroxine measurements during pregnancy. Thyroid.

[59] Bagis T, Gokcel A, Saygili ES (2001). Autoimmune thyroid disease in pregnancy and the postpartum period: relationship to spontaneous abortion. Thyroid.

[60] Dendrinos S, Papasteriades C, Tarassi K, Christodoulakos G, Prasinos G, Creatsas G (2000). Thyroid autoimmunity in patients with recurrent spontaneous miscarriages. Gynecol Endocrinol.

[61] Nambiar V, Jagtap VS, Sarathi V, Lila AR, Kamalanathan S, Bandgar TR (2011). Prevalence and Impact of Thyroid Disorders on Maternal Outcome in Asian-Indian PregnantWomen. J Thyroid Res.

[62] Kutteh WH, Yetman DL, Carr AC, Beck LA, Scott RT Jr (1999). Increased prevalence of antithyroid antibodies identified in women with recurrent pregnancy loss but not in women undergoing assisted reproduction. Fertil Steril.

[63] Bussen S, Steck T (1995). Thyroid autoantibodies in euthyroid non-pregnant women with recurrent spontaneous abortions. Hum Reprod.

[64] Pratt D, Novotny M, Kaberlein G, Dudkiewicz A, Gleicher N (1993). Antithyroid antibodies and the association with non-organ-specific antibodies in recurrent pregnancy loss. Am J Obstet Gynecol.

[65] Ashoor G, Maiz N, Rotas M, Jawdat F, Nicolaides KH (2011). Maternal thyroid function at 11-13 weeks of gestation and spontaneous preterm delivery. Obstet Gynecol.

[66] Muller AF, Verhoeff A, Mantel MJ, Berghout A (1999). Thyroid autoimmunity and abortion: a prospective study in women undergoing in vitro fertilization. Fertil Steril.

[67] Ghassabian A, Tiemeier H (2012). Is measurement of maternal serum TSH sufficient screening in early pregnancy? A case for more randomized trials. Clin Endocrinol (Oxf).

[68] Stagnaro-Green A, Roman SH, Cobin RH, el-Harazy E, Alvarez-Marfany M, Davies TF (1990). Detection of at-risk pregnancy by means of highly sensitive assays for thyroid autoantibodies. JAMA.

[69] Singh A, Dantas ZN, Stone SC, Asch RH (1995). Presence of thyroid antibodies in early reproductive failure: biochemical versus clinical pregnancies. Fertil Steril.

[70] Iijima T, Tada H, Hidaka Y, Mitsuda N, Murata Y, Amino N (1997). Effects of autoantibodies on the course of pregnancy and fetal growth. Obstet Gynecol.

[71] Poppe K, Glinoer D, Tournaye H, Devroey P, van Steirteghem A, Kaufman L (2003). Assisted reproduction and thyroid autoimmunity: an unfortunate combination. J Clin Endocrinol Metab.

[72] Marai I, Carp H, Shai S, Shabo R, Fishman G, Shoenfeld Y (2004). Autoantibody panel screening in recurrent miscarriages. Am J Reprod Immunol.

[73] Ghafoor F, Mansoor M, Malik T, Malik MS, Khan AU, Edwards R (2006). Role of thyroid peroxidase antibodies in the outcome of pregnancy. J Coll Physicians Surg Pak.

[74] Iravani AT, Saeedi MM, Pakravesh J, Hamidi S, Abbasi M (2008). Thyroid autoimmunity and recurrent spontaneous abortion in Iran: a case-control study. Endocrine Practice.

[75] Männistö T, Vääräsmäki M, Pouta A, Hartikainen AL, Ruokonen A, Surcel HM (2009). Perinatal outcome of children born to mothers with thyroid dysfunction or antibodies: a prospective populationbased cohort study. J Clin Endocrinol Metab.

[76] Soltanghoraee H, Arefi S, Mohammadzadeh A, Taheri A, Zeraati H, Hashemi SB (2010). Thyroid autoantibodies in euthyroid women with recurrent abortions and infertility. Iran J Reprod Med.

[77] Haddow JE, Cleary-Goldman J, McClain MR, Palomaki GE, Neveux LM, Lambert-Messerlian G (2010). Thyroperoxidase and thyroglobulin antibodies in early pregnancy and preterm delivery. Obstet Gynecol.

[78] Negro R, Schwartz A, Gismondi R, Tinelli A, Mangieri T, Stagnaro-Green A (2011). Thyroid antibody positivity in the first trimester of pregnancy is associated with negative pregnancy outcomes. J Clin Endocrinol Metab.

